# A Comparison in Travel Characteristics of Bike-Sharing between College Students and Office Workers Based on Theory of Planned Behavior

**DOI:** 10.3390/bs13040329

**Published:** 2023-04-13

**Authors:** Yuzhou Duan, Jiale Wang, Hui Li, Yibo Yan, Xu Zhang

**Affiliations:** College of Civil Engineering, Henan University of Technology, Zhengzhou 450001, China

**Keywords:** behavioral sciences, bike sharing, college students, office workers, theory of planned behavior, environmental awareness, travel behavior

## Abstract

As an important component of bike-sharing (BS) users, the travel behavior of college students and office workers is important to the promotion of BS within Chinese cities. To explore the influencing factors for the behavioral intentions of BS, this paper makes a different analysis between the two groups. Based on the theory of planned behavior, and using environmental awareness as an extended variable, a BS travel intention model was developed. A total of 676 valid questionnaires were collected and analyzed from college students and office workers in Zhengzhou. The results indicate that attitude, subjective norms, perceived behavior control, and environmental awareness have a positive impact on the behavioral intentions of BS. However, the influence degree of each variable is different between the two groups. Perceived behavior control, including travel time, travel cost, and cycling difficulty, has the greatest impact on BS behavioral intention for college students. Meanwhile, subjective norms, including policy and media publicity, has the most significant impact on BS behavioral intention for office workers. The impact of environmental awareness on college students’ use of BS is higher than that of office workers. We also found that undergraduates use BS more frequently than postgraduates. The findings provide the clear influence factors on behavioral intentions of BS between college students and office workers, that can help policy optimization in terms of bike-sharing systems, giving some suggestion for an approach devoted to deepen the individual-context interactions.

## 1. Introduction

With the rapid development of industrialization and urbanization, the continuous growth of motorized vehicles has led to an increasingly serious air pollution [1]. Transportation industry, the second largest carbon-emitting sector, accounts for approximately 23% of global carbon emissions [2,3]. In this context, the development of green transportation has become an effective measure to reduce air pollution and emissions [4]. Wherein BS has gained popularity in many cities for its green, low-carbon, and sustainability [5,6,7].

The first generation of BS system was born in the Netherlands, and was used for free. However, due to the lack of anti-theft devices, it went bankrupt. Then, the second generation of BS system became in Denmark, had features such as anonymous use, fixed lending and return points, as well as an anti-theft design. However, the fee problem led to many shared bicycles becoming ‘private’, so the system failed to become widespread. In the late 1990s, with the advanced electronic information and internet technology monitoring and managing BS in France [8], BS was widely promoted. Then, with the rise of dockless bicycle sharing in China in 2015, the number of BSs reached more than 23 million within two years. This type has rapidly spread and developed in many countries around the world [9,10,11,12].

As a common travel mode, BS is influenced by multiple factors [13]. In this research, we choose two types. The first type is individual attributes, such as age, income, gender, car ownership and other factors [14,15]. Another type is psychological factors, such as travel attitudes, subjective norms, perceptual behavioral control and environmental awareness [16]. Social psychologists believe that human behavior is driven by behavioral intentions, which vary with influencing factors [17,18,19,20,21]. Therefore, it is necessary to explore the psychological subjectivity and the perception of environmental factors that influence behavioral intentions for BS. In fact, there is the need to acquire knowledge about the life context of people that may influence attitudes and behaviour. BS is infact strongly influenced by the real opportunity to effectively commuting and travelling. Therefore there is the need to acquire multidimensional information related to the life context an individual psychological dimensions that may effect on the bicycle sharing behaviour. In this perspective the theory of planned behavior (TPB) can bring an added value, taking into consideration individual dimensions and their evaluation of the contextual feasibility of effective BS. Moreover it can support the strategies to make contextual feature more supportive for the sharing bicycle programs. This theory, proposed by Ajzen [22], consists of five latent variables, namely attitude, subjective norm, perceived behavioral control, behavioral intention and behavior. Each latent variable is determined by more than three observed variables. Behavioral intention reflects an individual’s willingness to perform a particular behavior, and influences decisions making directly. Whilst it is influenced by attitudes, subjective norms, and perceptual behavioral control. Perceived behavioral control and environmental awareness are the variable most strongly related to contextual variables as well as behaviour. In this vision taking into account the theory of planned behaviour may help in the understanding of the individual-context related variables. 

From the perspective of BS user groups, it have mainly concentrated on college students and office workers in China [23]. For one thing, the BS increase the interest of college students, with the advantages of easy cycling, low costs and low carbons [24]. Furthermore, it could reduce the travel pressure on campus as an alternative means [25]. Over 33.6% of college students have already used BS [26]. For another, BS is seen as a convenient and effective way to solve the ‘last mile’ problem for office workers. [27,28]. However, considering the influence factors on travel behavior will vary with individual attributes, there is a lack of the travel behavior differences between college students and office workers groups. To explore this issue, this paper add the environmental awareness to extends the TPB model, and then constructs a structural equation model. Taking Zhengzhou city as an example, a questionnaire survey will be conducted to college students and office workers. Then, we will explore the influence degree of environmental awareness on BS travel behavior choices. The combined effect coefficients of attitudes, subjective norms, perceived behavioral control and environmental awareness on behavioral intentions, will be explored. And the differences between college students and office workers will also be analyzed. 

The study is organized as follows: Section 2 summarizes the research methods and influencing factors of travel behavior intentions. Section 3 explains the theoretical basis, modeling process, and research methods. Section 4 provides the survey process and the analysis of the survey data. Section 5 selects actual case data for analysis and presents the results. Section 6 analyzes and discusses the results of the case study. Section 7 is the research conclusion and limitations.

## 2. Literature Review

### 2.1. Theory of Planned Behavior

Currently, research on transportation mode choice mainly consists of two methods: the aggregate model and disaggregate model. Wherein, the disaggregate model has a wide range of applications in the field of travel modes. Gebhart et al. analyzed the use of BS in Washington and found that factors such as low temperature and rainfall reduced the possibility of using BS, while good weather increased users’ willingness to choose BS travel [29]. Li et al. pointed out that air pollution has a significant negative effect on the users’ willingness to use BS [30] while reducing pickup time and travel costs can promote the use of BS [31]. The utility function in the disaggregate model does not consider the psychological factors that influence behavior, but the influence that psychological factors have on residents’ travel behavior cannot be ignored, and some theories that consider psychological factors are widely used. TPB based on the covariance matrix of variables to analyze the relationship between variables is one of the important tools of multivariate data analysis, which can effectively deal with the relationship between potential variables (psychological, educational, social, and other concepts) and measurement variables, and provide theoretical support for studying the psychological characteristics of travel behavior [22]. Jing et al. studied the influence of psychological latent variables on school commute mode choice based on an extended TPB model [32]. Hu introduced individual attributes, travel environment, and travel characteristics variables based on the TPB model and analyzed the dependency effect of public transportation [33]. Chen et al. expanded the TPB and proposed that perceived benefits and government policies are important factors in increasing college students’ willingness to use BS [34]. Ju et al. used the technology acceptance model and TPB as frameworks and introduced two variables, government policies and travel behavior, to analyze the factors influencing the intention to use car-sharing [35].

### 2.2. Application of TPB in BS

TPB consists of five components: attitude (AT), subjective norm (SN), perceived behavioral control (PBC), behavioral intention (TBI), and behavior (TB). According to Ajzen, AT refers to an individual’s evaluation of specific behavior, which determines their preference for a product or service. SN refers to the individual social pressure when adopting a certain behavior. Support or approval from family and friends increases the individual’s willingness to adopt the behavior, while the opposite is also true. PBC reflects the relationship between existing resources and expected barriers. The more resources an individual has, the fewer expected barriers there are, and the greater the influence of PBC on TBI [22]. TPB has been applied in many fields, including transportation, population movement, and consumer motivation [36,37,38]. Some studies have used TPB to analyze the willingness of individuals to use BS for transportation.

#### 2.2.1. Attitude

Attitude (AT) refers to the evaluation and conceptualization of an individual’s attitude towards a specific behavior, which determines their degree of preference for a product or service. In the context of BS travel behavior, AT can be the individual’s perception and evaluation of BS travel [39]. Previous studies have confirmed that AT has a significant positive impact on BS travel behavior [40]. Sun investigated the effect of AT on civilized cycling intentions and actual behavior based on the TPB and found that AT is positively related to both users’ civilized cycling intentions and their actual behavior [39]. Ransford found that for office workers who use bicycles for work, AT does not significantly affect behavioral intentions [41]. Research findings that in the E-bike-sharing travel model, the influence of AT on travel intentions is less significant than that of SN and PBC [42].

#### 2.2.2. Subjective Norm

Subjective norm (SN) refers to the social pressure that individuals face when adopting a certain behavior, where support or approval from family and friends can increase willingness to adopt a certain behavior, and vice versa [19]. Regarding bicycle travel, parents may not recommend to their children to ride bicycles due to safety concerns, while friends and classmates may use the advantages of bike sharing to increase their willingness to travel [42]. Previous studies have demonstrated the positive impact of bike sharing on the willingness to travel by bicycle, with some scholars arguing that the influence of SN on the willingness to use bike sharing is most significant [42,43,44]. Measures such as policy guidance, environmental awareness, and price incentives can have a positive impact on bicycle travel through SN.

#### 2.2.3. Perceptual Behavioral Control

Perceptual behavioral control (PBC) reflects the relationship between existing resources and expected obstacles. A worse environment will increase the expected obstacle [44,45]. The more resources an individual has, the fewer expected obstacles there are, and the greater the impact of PBC on TBI [46]. In this study, if users have proficient cycling stills and the BS environment is good enough, their intentions to use BS will increase. Factors such as travel cost, membership card, and driving difficulty all have an impact on PBC. The stronger the PBC, the more likely residents are to use BS [47,48].

#### 2.2.4. Environmental Awareness

According to Ajzen, the TPB can be extended when other variables are found to have important implications for the original structure [49]. Environmental awareness (EA) has been shown to have a significant impact on people’s transportation choices [50,51]. Groot added environmental awareness variables under the TPB framework to verify the hypothesis that environmental awareness has a direct impact on behavioral intentions [52]. Chen took environmental protection awareness as an expansion variable, built a public transport travel choice behavior model, and compared and analyzed the quantitative relationship between various influencing factors of public transport travel choice behavior [53]. The results show that environmental protection awareness variables have a positive impact on public transport travel choice behavior. Some scholars have also explored the relationship between environmental awareness and BS usage and found that the willingness to use BS is enhanced by low-carbon travel and environmental awareness [54,55]. Therefore, this study includes EA as an expanded variable in the TPB structure.

### 2.3. Summary

Based on a review of the literature, the applicability of TPB’s analysis of BS travel behavior has been confirmed, and AT, SN, and PBC have a significant positive impact on BS travel willingness. However, the current research in the field of BS travel behavior still has the following problem: there are few studies on the differences among BS user groups. As the two most important user groups in China, whether there are differences in the characteristics of BS travel behavior between college students and office workers, lacks clear experimental results.

To fill these research gaps, this study considers the impact of individual and psychological factors on BS travel behavior and makes a comparative study of college students and office workers. This study incorporates EA variables into TPB theory, and builds BS travel behavior choice model. Then, we conduct a questionnaire survey on the individual attribute. Finally, we analyze the impact of psychological factors on BS travel intentions, and make a comparison between college students and office workers.

## 3. Methodology

### 3.1. Theoretical Applicability

**Figure 1:**
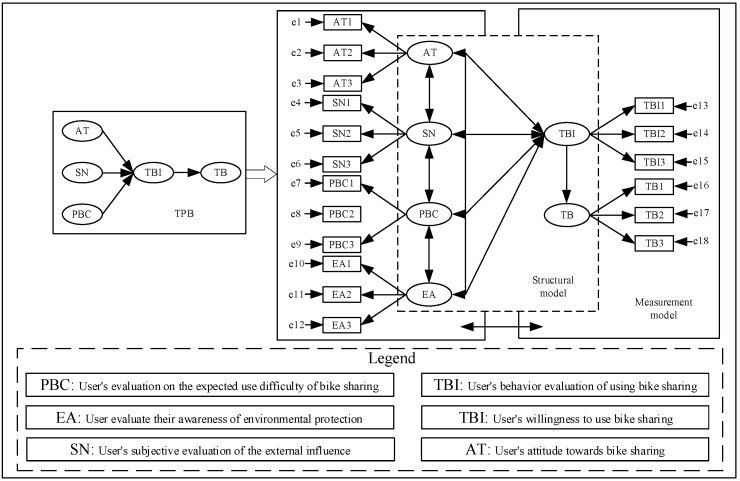
Travel behavior model. TBI repeted in the legenda.

In studying the choice factors of shared bicycle behavior intention, many variables are not directly observed. The structural equation model (SEM) can deal with the problems between multiple dependent variables. Based on the TPB theoretical framework, adding EA as a potential variable, the explicit characteristics of EA variables are evaluated through subjective feelings, such as environmental awareness self-evaluation, environmental behavior evaluation, and the impact of the environmental publicity, and a structural equation model of BS travel behavior is constructed (as shown in Figure 1. By comprehensively considering variables, such as AT, SN, PBC, and EA, the significance of TBI and TB were detected. Then, we analyzed the travel choice behavior combined with the survey data. The model framework and variable definition description are shown in Figure 1. 

### 3.2. Theoretical Hypothesis

**Figure 2:**
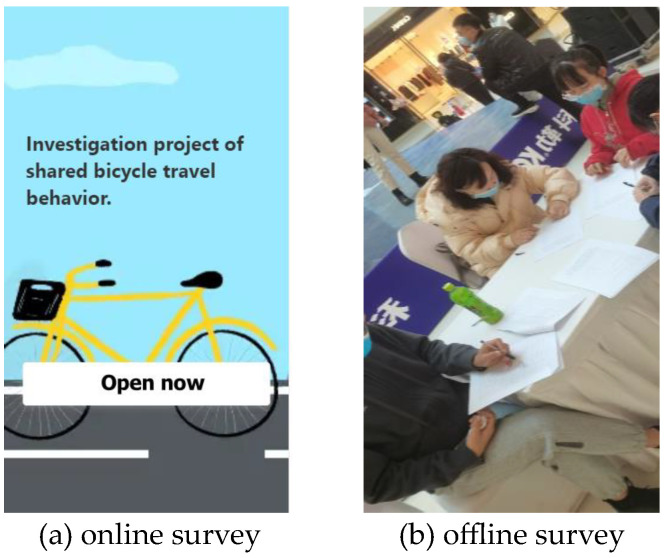
Investigation process.

We make a hypothesis about the path relationship of the variables in the model. The specific assumption path is shown in Figure 2 (H1 is AT pointing to TBI, H2 is SN pointing to TBI, H3 is PBC pointing to TBI, H4 is EA pointing to TBI, H5 is TBI pointing to TB):

**Hypothesis 1:**  *AT has a positive impact on the behavior choice of BS.*

**Hypothesis 2:**  *SN has a positive impact on the behavior choice of BS.*

**Hypothesis 3:**  *PBC has a positive influence on the behavior selection of BS.*

**Hypothesis 4:**  *EA has a positive impact on the behavior choice of BS.*

**Hypothesis 5:**  *TBI has a positive impact on the behavior choice of BS.*

### 3.3. Model Building

To describe the causality between variables more directly, we have established a mathematical model to analyze the SEM. Where X is an explicit exogenous variable, Y is an explicit endogenous variable, ξ is an exogenous latent variable, and η is an endogenous latent variable. Observed variables can be directly observed or measured compared to ‘latent variables’. Latent variables are variables that cannot be measured directly in practical work, including abstract concepts and variables that cannot be accurately measured for various reasons. The measurement model is used to describe the relationship between the manifest variables X, and Y, and the latent variables ξ and η and the structural model are used to describe the relationship between the latent variables ξ and η.

#### 3.3.1. Measurement Model

(1) $$\underset{\left(12\times 1\right)}{X_{}}=\underset{\left(12\times 4\right)}{E_{X}}\underset{\left(4\times 1\right)}{\xi_{}}+\underset{\left(12\times 1\right)}{\delta_{}}$$

(2) $$\underset{\left(2\times 1\right)}{Y_{}}=\underset{\left(2\times 6\right)}{E_{Y}}\underset{\left(6\times 1\right)}{\eta_{}}+\underset{\left(2\times 1\right)}{\varepsilon_{}}$$

where X consists of 12 measured variables of the dependent variable, ξ consists of 4 potential exogenous variables, $E_{X}$ denotes a X-to-ξ regression coefficient or factor compliance matrix, δ is a vector composed of the observation errors of X; Y consists of the 6 measured variables of the dependent variable, η consists of 2 potential endogenous variables, $E_{Y}$ denotes a Y-to-η regression coefficient or factor compliance matrix, and ε is a vector composed of the observation errors of Y.

#### 3.3.2. Structural Equation Model

(3) $$\underset{\left(2\times 1\right)}{\eta_{}}=\underset{\left(2\times 2\right)}{B_{}}\underset{\left(2\times 1\right)}{\eta_{}}+\underset{\left(2\times 4\right)}{F_{}}\underset{\left(4\times 1\right)}{\xi_{}}+\underset{\left(2\times 1\right)}{\zeta_{}}$$

where *B* is the structural coefficient matrix of the endogenous latent variable, whose diagonal element is 0. The matrix determinant of B is required not to be 0. The *B* coefficient matrix reflects the factor load matrix of the model’s endogenesis latent variable to other. F is the coefficient matrix of ξ to η. ξ is the error vector of the model [16,22].

### 3.4. Model Solving

Firstly, SPSS is used to digitally process the results of the questionnaire. The data were then classified and tested by reliability and factor analysis. Then, the data results are calculated in the model framework designed by AMOS to test the suitability of the model and the rationality of the structural design. Finally, the path regression coefficient between each variable is calculated.

## 4. Questionnaire Survey and Results

### 4.1. Questionnaire Design

Our questionnaire is designed with the Likert five-level scale. The questionnaire is divided into two parts. The first part investigates the distribution of individual attributes, and the second part investigates the public’s attitude toward BS travel behavior. According to the needs of the model, we have designed six sections as follows: AT, SN, PBC, EA, TBI, and TB. The extension variable of TPB is EA (as shown in Table 1).

To clarify the mediating effect of potential variables on behavior, this paper evaluates the AT, SN, PBC, EA, TBI, and TB of BS travel by measuring variables. In this survey, AT refers to the user’s preference for BS travel reflected by this behavior, which was evaluated through the three measurement variables of travel interest, convenience, and daily maintenance. A positive evaluation would improve travel attitude towards BS. SN refers to the social pressure on users when using BS for travel. This paper measures social pressure from three perspectives: policy support, media publicity, and suggestions from relatives and friends. The positive pressure will have a positive impact on SN. This paper evaluates PBC by taking cycling difficulty, travel cost, and travel time as measurement indicators. As an expansion structure, EA reflects the impact of environmental awareness on users’ BS travel behavior. It investigates and analyzes the frequency of users’ participation in environmental activities and the environmental evaluation of BS travel, and calculates the function coefficient of environmental awareness variables. The TBI sets three questions to measure: “Fixed parking has a great impact on my choice of BS”, “The willingness to share bicycles is higher than in other modes of transportation”, and “Willing to apply for bike sharing a discount card”. These reflect the respondents’ inclination to BS travel behavior, and explain and predict the actual behavior of individuals. TB represents the result of the individual’s actual action.

### 4.2. Data Collection

In this study, the campus of the Henan University of Technology and the central area of Zhengzhou city, including Zhongyuan District, Erqi District, Jinshui District, Guancheng District, and Huiji District, were used as the survey sites for the questionnaire survey. The surveys were conducted from November 2021 to January 2022, both online and offline (Figure 2a shows the online survey, and Figure 2b,c shows the offline survey). Schools, parks, shopping malls, and office buildings were the main places for questionnaire distribution. 

### 4.3. Distribution of Respondents

**Figure 3:**
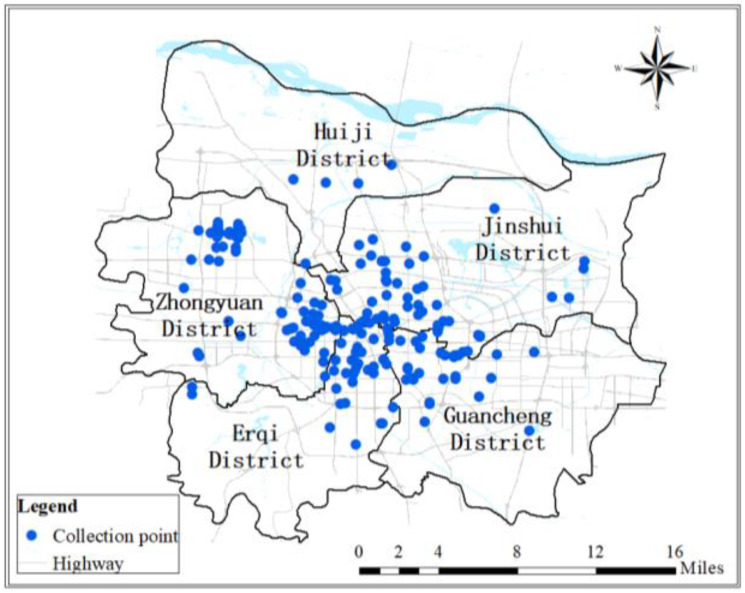
Location distribution of survey objects.

After two months of investigation, a total of 900 questionnaires were issued; then, 676 valid questionnaires were collected, including 427 campus questionnaires and 249 off-campus questionnaires, which meet the basic requirements for AMOS [16,22]. Figure 3 shows the distribution of respondents.

**Figure 4:**
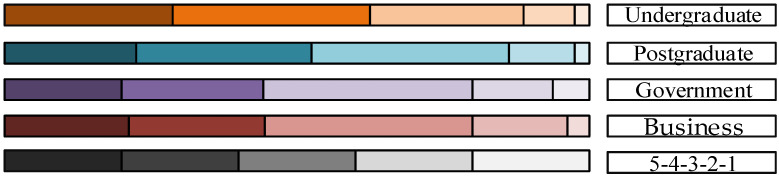
Individual attribute difference in BS travel willingness.

Table 2 shows the distribution characteristics of the individual attributes of the survey samples. In accordance with the distribution of individual attributes, college students were divided into undergraduate and postgraduate, and office workers were divided into government personnel and business personnel. The cross-analysis of the individual attributes and BS travel intention was carried out. As shown in Figure 4, the color depth corresponds to 5-1 points of the questionnaire answer, reflecting the degree of BS travel willingness(the yellow bar chart represents undergraduate students, the blue bar chart represents postgraduates, the purple bar chart represents government workers, the red bar chart represents business personnel). The results show that the willingness of college students and office workers to use BS is more than 50%, the willingness of college students to use BS is slightly higher than that of office workers, and the willingness of postgraduates to use BS is lower than that of undergraduates.

### 4.4. Data Analysis

#### 4.4.1. Questionnaire Data Test

Before the path regression coefficient analysis, the quality of the questionnaire design and survey results were tested to ensure that the data collected in the questionnaire could meet the requirements of SEM analysis and meet the consistency and reliability of the results. To achieve the above requirements, the reliability of each dimension of the questionnaire was tested through a parameter named Cronbach’s alpha (α). If it is greater than 0.6, we can conclude that the questionnaire meets the requirements, otherwise, the internal consistency reliability is insufficient. On this basis, the average variance extracted (AVE) values was used to test the classification of the questionnaire and the quality of data filling. If AVE is greater than 0.4 means the measurement results can reflect the selection degree of the investigated content meets the requirements. That is, the data quality of the questionnaire that was collected is meets the standards. In Table 3, we can see that in the reliability test, the PBC of the questionnaire in the school α value is 0.57, slightly less than 0.6, which proves that the reliability of the answer is high, and the investigators’ choice of the same question has a low systematic error, ensuring the consistency, stability, and reliability of the test results. In the validity test, the value of AVE is greater than 0.4, that is, the answer sheet data, to a large extent, reflect the accurate content of the questions investigated. On this basis, factor dimension reduction analysis was carried out through SPSS, in which the value of significance (Sig) was 0, which met the significant requirements of the test results. When the cumulative value of the total variance is greater than 60%, the number of principal component clusters of the problem is 6, which is consistent with the 6 aspects of the model framework and meets the model operation standard.

#### 4.4.2. Structural Parameter Estimation

The questionnaire questions are classified through dimension reduction analysis, and the significance of the parameters of each dimension (AT, SN, PBC, EA, TBI, and TB) is estimated. The parameter significance estimation (*p*) represents the parameter estimation value under non-standardization. If *p* is less than 0.05, it is significant, and if *p* is less than 0.1, it is marginally significant, and the load coefficient should be greater than 0.4. As shown in Table 4, except for the load coefficient of EA2 in the office workers group, which is slightly less than 0.4, the other indicators meet the requirements, and the significance of this coefficient is strong, which proves this proves that the model has good explanatory power [16,22].

## 5. Case Study

### 5.1. Model Calibration

To verify the matching degree between the evaluation data and the hypothesis model, the fitness test of the constructed structural equation model was conducted first. Model test indicators contain absolute goodness-of-fit test indicators, including the root mean square of the approximation error (RMSEA) and goodness-of-fit index (GFI), value-added goodness-of-fit test indicators, including normative fitting index (NFI), comparative fitting index (CFI), value-added fitting index (IFI), and degree of freedom of adjustment goodness-of-fit index (AGFI). Among them, the requirements of each index are: the RMSEA should be less than 0.08, CFI, GFI, and IFI should be greater than 0.9, and the other indexes should be greater than or close to 0.85 [15,54]. As shown in Table 5, all indicators meet the standard requirements, the fitness index of the model meets the test standard, and the standardized path coefficient can be analyzed [16,22,56].

### 5.2. Model Path Analysis

**Figure 5:**
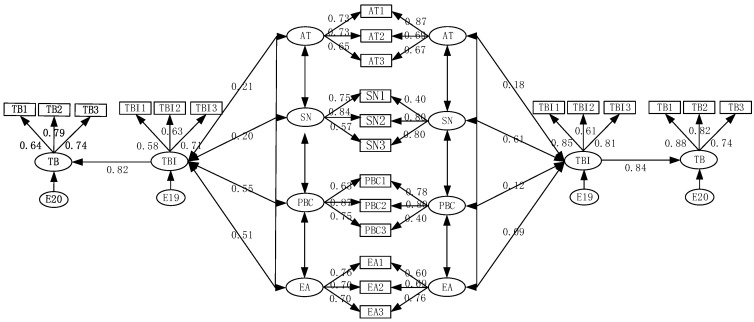
Model results—college students (**left**)/office workers (**right**).

Through the model analysis of AMOS, it can be concluded that the impact of environmental awareness on BS travel behavior intention of office workers is insignificant. Additionally, the *p*-value is 0.266, which indicates that the original hypothesis is not tenable. AT has a positive impact on the TBI of college students, but its significance is very low. All other assumptions are valid. (see Table 5). It can be seen in Table 5 and Figure 5 that the influence coefficient of TBI on TB is 0.84. PBC and EA have the most significant impact on the BS travel behavior willingness of college students. AT, SN, PBC, and EA have a positive impact on TBI, with influence coefficients of 0.15, 0.10, 0.46, and 0.36, respectively. SN has the most significant impact on the BS travel behavior willingness of office workers. The impact coefficients of AT, SN, and PBC on TBI are 0.18, 0.61, and 0.09, respectively.

## 6. Results and Discussion

As public transportation, BS has been promoted worldwide because of its green, low-carbon, flexible, and convenient advantages [4,5]. Previous studies have shown that guiding the sustainable development of travel modes can reduce the pollution and consumption caused by motor vehicle travel [32,33,34]. In China, college students and office workers are the main user groups of BS, but there are few studies on the differences in BS travel behavior between these two groups. To solve this problem, this paper studies the factors that affect BS travel based on the TPB and cross analysis method.

As the basic structure of TPB, AT, SN, and PBC have a significant impact on TBI. This conclusion has been confirmed in the application of BS [44,48,53]. This paper also draws a similar conclusion. However, in the study of the differences between college students and office workers, we have confirmed that there are individual differences in the degree of influence between various variables.

Among the variables that affect college students’ BS travel intention, SN and PBC have a significant positive impact on TBI, and PBC has the greatest impact on TBI. Among the three measures of SN, the support and encouragement of relatives and friends have the greatest impact on BS travel attitudes. The standardized path coefficient is 0.84. The policy has the smallest impact, and the path coefficient under standardization is 0.57. The three measurement variables of PBC have significant positive effects on PBC, and the standardized path coefficient is between 0.63 and 0.87. Driving difficulty, use cost, and travel time are the main factors that affect college students’ choice of BS travel. Among them, the use cost has the greatest impact on a student’s choice of BS travel, with an impact coefficient of 0.86. The direct impact of AT on TBI is not significant, which is consistent with the conclusion of Acheampong [41]. Compared with interest and vehicle maintenance issues, travel costs, travel time, and convenience are concerns for college students [47]. Operating enterprises should increase the preferential strength of campus BS, and drive the college students’ willingness to use BS through price incentives. Relevant operators can innovate marketing strategies based on this, such as group purchase discounts, one card for multiple purposes, etc. At the same time, we can cooperate with school associations or other units to increase the publicity of campus BS, and enhance the effect of SN on TBI.

Among the variables that affect the travel intention of office workers, AT, SN, and PBC have a significant positive impact on TBI, with SN having the largest impact on TBI and PBC having the smallest impact on TB. Unlike college students, “media publicity” and “policy guidance” have a greater impact on the choice of BS travel by office workers [53,54], which may be due to the proportion of government staff among respondents. PBC has a positive impact on TBI, but its significance is lower than that of college students.

Research has proved that EA has a positive impact on BS’ willingness to travel [57,58]. In this paper, EA is included in TPB as an expansion variable to analyze college students and office workers, respectively. It was found that in the travel model of the two groups, the three measured variables have positive effects on EA, and the standardized path coefficient is between 0.60 and 0.78. The impact of EA on college students’ choice of BS travel is greater than that of office workers. Operating enterprises can increase environmental protection publicity activities and advocate for green travel for college students. In addition, the production and delivery of BS should meet actual needs to avoid resource wastage. 

In addition, we conducted statistics on the distribution of personal attributes in the survey results. Similar to the proportion of BS users in China [24], the frequency of BS trips was above 50% for both college students and office workers, with college students using BS slightly more frequently than office workers. Undergraduates used BS more frequently than postgraduates, who had regular weekly classes and commuted more frequently between different academic buildings compared to postgraduates.

## 7. Conclusions

To explore the relevant factors that affect users’ BS travel intention, this paper analyzes the differences between college students and office workers, establishes a BS travel behavior model based on TPB and expansion structure, and analyzes the significance of five standard structures and one expansion structure. To ensure the authenticity of the experimental conclusion, we conducted a questionnaire survey on the college students of the Henan University of Technology in Zhengzhou and the office workers in the Zhengzhou city center and designed and investigated the seven variables of BS travel (attitude, subjective norms, perceptual behavior control, environmental awareness, behavioral willingness, and behavior). The contributions of this study are as follows:

(1) Taking the campus of the Henan University of Technology and the central area of Zhengzhou city as the survey sites, the questionnaire was designed using the Likert five-level scale method, and the reliability and validity of the questionnaire were tested. The recovered data can meet the requirements of SEM analysis, and ensure the consistency and reliability of the results. At the same time, this paper analyzes the individual differences in respondents’ BS travel willingness and explores the differences in BS travel willingness among undergraduates, graduate college students, business personnel, and civil servants;
(2) Constructing the BS travel choice willingness model based on the theory of planned behavior, with “environmental awareness” as the core variable. After testing, the model meets the requirements, and the fitting degree is good;
(3) This paper analyzes the differences in BS travel behavior between college students and office workers, explores the impact of AT, SN, PBC, and EA on TBI from the psychological factors, and analyzes the impact of travel cost, travel time, convenience, policy, driving difficulty and other factors on college students and office workers’ choice of BS travel.
(4) According to Orford [59], the community psychology seminal author, behaviour is the effect of the equation Person X Context. In this sense this article proposes the use of TPB as a further perspective to understand the complex interactions among individuals and contexts, that will enrich the understanding of the individual behaviours in social contexts. Variables at play are individuals, but they are influenced by organizational and societal determinants.

These conclusions can provide theoretical guidance for the operation of BS enterprises on university campuses, and corresponding improvement measures can be made accordingly. However taking into consideration community psychology approach it is worth to consider thath “The emphasis of community psychology in practice is on prevention, intervention and policy change at a non-individual level, rather than on treatment at the personal level. In order to promote individual and collective health and well-being and to reduce distress and difficulties, it is necessary to promote change in the social, economic and environmental arrangements that give rise to such problems [59].” This is the community psychology lesson and the PBT is in it self lacking for a more political vision.

In fact this research also has the following shortcomings: (1) In addition to the psychological factors, many studies have found the significant effect of the built environment on BS usage; further research should consider this factor [57,58,60]; (2) This study does not consider the difference between BS and other means of transportation, such as private bicycles and scooters (electric scooters have been widely used in many countries [61]). This is also the direction of our further research, encompassing also a perspective able to act at a non individual level.

## Figures and Tables

**Table 1:** Description of model variables.

Variable	Symbol	Description
AT	X1	I’m interested in using BS.
	X2	I think it’s convenient to use BS to travel on campus.
	X3	Don’t worry about vehicle maintenance.
SN	X4	Media promotion.
	X5	Opinions of relatives and friends.
	X6	Policies issued by the local government.
PBC	X7	Easy cycling.
	X8	Low costs.
	X9	Choose BS when travel time is limited.
EA	X10	Environmental protection publicity activities will promote the use of shared bicycles.
	X11	I often participate in environmental activities.
	X12	Using BS can improve resource utilization.
TBI	Y1	Fixed parking has a great impact on my choice of BS.
	Y2	The willingness to share bicycles is higher than in other modes of transportation.
	Y3	Willing to apply for BS a discount card.
TB	Y4	I often choose BS for travel.
	Y5	I will continue to use BS for travel.
	Y6	I often use BS to go to work/school.

**Table 2:** Demographic characteristics in this survey.

College Students				Office Workers			
Characteristics	Constructs	Number	Ratio (%)	Characteristics	Constructs	Number	Ratio (%)
Gender	Male	227	53.2	Gender	Male	100	40.2
	Female	200	46.8		Female	149	49.8
Grade	Freshman	82	19.2	occupation	Business	74	29.7
	Sophomore	94	22.1		Service	57	22.9
	Junior	89	21.0		Public Institution	68	27.3
	senior	85	19.7		Government	50	20.1
	Postgraduate	77	18.0				

**Table 3:** Model parameter estimation.

Variable	Title	College Students				Office Workers			
		*p*	Path Coefficient	α	AVE	*p*	Path Coefficient	α	AVE
AT	AT1-3	<0.01	0.730/0.731/0.651	0.798	0.516	<0.01	0.686/0.669/0.871	0.839	0.559
SN	SN1-3	<0.05	0.750/0.845/0.571	0.722	0.576	<0.05	0.798/0.796/0.371	0.819	0.469
PBC	PBC1-3	<0.01	0.756/0.859/0.632	0.570	0.570	<0.01	0.760/0.693/0.600	0.847	0.473
EA	EA1-3	<0.01	0.685/0.694/0.780	0.728	0.520	<0.05	0.780/0.334/0.799	0.770	0.453
TBI	TBI1-3	<0.01	0.717/0.620/0.568	0.807	0.407	<0.01	0.812/0.614/0.849	0.673	0.586
TB	TB1-3	<0.01	0.740/0.789/0.645	0.875	0.529	<0.01	0.744/0.824/0.822	0.763	0.636
Sig			0				0		
Explain total variance			69.865				69.151		
Number of clusters			6				6		

**Table 4:** Structural fitting test results.

Measurement	X^2^	df	RMSEA	CFI	NFI	GFI	IFI	AGFI
Standard value	-	-	<0.1	≥0.90	≥0.85	≥0.90	≥0.90	≥0.85
Actual value (college students)	381.48	124	0.07	0.93	0.90	0.91	0.93	0.88
Actual value (office workers)	233.98	124	0.06	0.94	0.90	0.90	0.94	0.87

**Table 5:** Hypothesis test results.

	College Students			Office Workers		
Path	Path Coefficient	*p*	Hypothesis	Path Coefficient	*p*	Hypothesis
H1	0.15	0.170	Not supported	0.18	**	supported
H2	0.1	**	supported	0.61	***	supported
H3	0.46	***	supported	0.09	*	supported
H4	0.36	***	supported	0.12	0.278	Not supported
H5	0.84	***	supported	0.84	***	supported

Notes: ***: *p* < 0.001, **: *p* < 0.0 5, *: *p* < 0.1.

## Data Availability

Not applicable.
